# Characterizing uncertainty in goals-of-care discussions among black and white patients: a qualitative study

**DOI:** 10.1186/s12904-022-00912-9

**Published:** 2022-02-17

**Authors:** Annie T. Chen, Shelley Tsui, Rashmi K. Sharma

**Affiliations:** 1 Department of Biomedical Informatics and Medical Education, University of Washington School of Medicine, 850 Republican St, Box 358047, 98109 Seattle, WA United States; 2 University of Washington, WA Seattle, United States; 3 Division of General Internal Medicine, University of Washington School of Medicine, Seattle, WA United States

**Keywords:** Uncertainty, Information needs, Goals-of-care discussions, Communication, Racial differences

## Abstract

**Background:**

Uncertainty has been associated with distress and poorer quality of life in patients with advanced cancer. Prior studies have focused on prognostic uncertainty; little is known about other types of uncertainty that patients and family members experience when discussing goals of care. Understanding the types of uncertainty expressed and differences between Black and White patients can inform the development of uncertainty management interventions.

**Methods:**

This study sought to characterize the types of uncertainty expressed by Black and White patients and family members within the context of information needs during inpatient goals-of-care discussions. We performed a secondary analysis of transcripts from 62 recorded goals-of-care discussions that occurred between 2012 and 2014 at an urban, academic medical center in the United States. We applied an adapted taxonomy of uncertainty to data coded as describing information needs and used an inductive qualitative analysis method to analyze the discussions. We report the types of uncertainty expressed in these discussions.

**Results:**

Fifty discussions included patient or family expressions of information needs. Of these, 40 discussions (n=16 Black and n=24 White) included statements of uncertainty. Black and White patients and families most frequently expressed uncertainty related to processes and structures of care (system-centered uncertainty) and to treatment (scientific uncertainty). Statements of prognostic uncertainty focused on quantitative information among Whites and on qualitative information and expectations for the future among Blacks.

**Conclusions:**

Black and White patients and families frequently expressed system-centered uncertainty, suggesting this may be an important target for intervention. Addressing other sources of uncertainty, such as prognostic uncertainty, may need more tailored approaches.

## Background

Patients with advanced cancer and their families frequently face high-stakes decisions about treatment options and goals of care in the hospital [1–3]. Given advances in cancer treatment, patients and families must understand complex information about treatment options, disease severity, and prognostic uncertainty to engage effectively in shared decision making. Unfortunately, studies have found patients frequently demonstrate poor understanding of prognosis and likelihood of cure, [4–7] desire more information about prognosis, [8–10] and report additional information needs [11]. In addition, patient, family, and clinician perceptions of information exchange are often discordant, with clinicians often underestimating patients’ information needs and overestimating their understanding and awareness of information [12].

A key challenge that patients, families, and clinicians face in discussing goals of care is addressing the uncertainty that comes with illness [13, 14]. This uncertainty may be described in various ways, including as a stressor, a perceived loss of control, and a state of doubt or not knowing [15]. Within the context of serious illness, uncertainty can manifest in situations involving ambiguity, inconsistency, vagueness, unpredictability, lack of information, unfamiliarity, and complexity [16]. Patients also report experiencing uncertainty about various domains including work, finances, family life, and the future. [17] While patients’ responses to uncertainty can depend on their engagement, temporal focus, and information preferences, [18] different types of uncertainty may also warrant different management strategies. [19] Previous studies have focused on prognostic uncertainty, [20, 21] which has been associated with lower quality of life and higher levels of distress for patients [20, 22]. However, research on other types of uncertainty, which may also be important sources of distress for patients with advanced cancer, remains limited.

To our knowledge, though the types of uncertainty expressed by clinicians in the context of advanced cancer has been examined, [23] similar work has yet to be conducted on the uncertainty expressed by patients. In this study, we adapt a synthesis of uncertainty taxonomies [19] to the palliative care context to examine uncertainty as expressed in goals-of-care discussions involving patients, family members, and clinicians. We chose to focus on expressions of uncertainty in the context of information needs to identify content areas to address in communication-based interventions. In addition, given the well-documented racial and ethnic differences in patient-family-clinician communication, [24–30] we set a secondary goal of comparing the ways in which uncertainty is expressed by patients and family members in discussions with Black and White patients. This work can inform the development of interventions to support patients, families, and clinicians in addressing uncertainty.

## Methods

### Study design

This study involved secondary analysis of data collected in an observational study of goals-of-care discussions at a National Cancer Institute-designated cancer center between physicians and patients with advanced cancer from October 2012 and November 2014 [31]. Goals-of-care discussions were considered to be discussions in which clinicians planned to discuss goals of care, prognosis, end-of-life decision-making, advance care planning, hospice, or to break bad news to inform preference-sensitive treatment decisions.

### Participants

Participants were recruited from inpatient palliative care consult, oncology, and hospitalist-oncologist co-managed services. Eligible patients had to speak English, be 18 years of age or older, have metastatic cancer that had progressed despite treatment, and be mentally and physically able to communicate about their care. A research assistant identified planned goals-of-care discussions through chart review and communication with medical teams.

### Data preparation and analysis

The dataset included patients’ demographics (e.g., self-reported race, age, sex), and their disease characteristics. Discussions were audio recorded, transcribed verbatim, and de-identified. Our analysis was conducted in two phases. The first phase has been previously described [31] and utilized an inductive coding approach [32, 33] to study four stages of decision making: information exchange, deliberation, making a patient-centered recommendation, and wrap up of decisional status. In the second phase, we examined data that had been coded as pertaining to information needs, one of our emergent themes, to characterize the nature of uncertainties underlying information needs. To do this, we reviewed literature related to uncertainty [20, 34, 35] to adapt an existing taxonomy of uncertainty in healthcare [19] to the serious illness context. One author (ST) coded all of the information needs segments to identify the type of uncertainty expressed, if any. Then the other two authors (RKS and ATC) reviewed the assignments, and all three authors discussed codes for which there might be a differing opinion and reconciled as needed. In comparing the nature of uncertainty in discussions involving Black and White patients, we considered both the frequency of codes and the nature of the ideas/questions being expressed.

As Braun and Clarke write, “more instances do not necessarily mean the theme itself is more crucial”(p. 82) [36]. In the selection of themes to describe, we considered factors including the theme frequency, how many people mentioned it, how it was expressed, and contextual factors relating to the healthcare system. We believe that reporting theme frequency would obfuscate the nuanced nature of this analysis and thus have chosen not to do so. However, as we recognize that frequency, though not the sole indicator, is one indicator of importance, in our reporting we provide linguistic cues such as ‘more’ or ‘less’ common to guide the reader with a general sense of prominence within the dataset.

### Ethical considerations

 Eligible patients were approached for consent prior to participation in the discussions; all persons who participated in the discussions provided informed consent. The study was approved by the Northwestern University Institutional Review Board and all study procedures complied with human subjects regulations.

## Results

### Sample

Of the 62 recorded discussions, 50 included discussion of information needs among Black (*n* = 19) or White (*n* = 31) patients. Demographics for the patient participants for these discussions are displayed in Table 1. Family members were present in 74% (*n* = 14) of discussions with Black patients and 68% (*n* = 21) of discussions with White patients.

**Table 1:** Sample Characteristics

Characteristics	Black Patients (*N *= 19)	White Patients (*N *= 31)
Age years, mean (SD)	59.7 (12.2)	60.4 (13.0)
Female sex, n (%)	9 (47%)	15 (48%)
Education, ≤ High school	11 (58%)	5 (16%)
Type of cancer		
Hematologic	2 (11%)	4 (13%)
Lung	6 (32%)	4 (13%)
Pancreatic	5 (26%)	6 (19%)
Gastrointestinal (e.g., colorectal, liver)	2 (11%)	5 (16%)
Other (e.g., renal, breast, melanoma)	4 (13%)	12 (39%)
Family present, n (%)	14 (74%)	21 (68%)
Participating clinicians^a^		
Palliative care attending	14 (74%)	27 (87%)
Palliative care fellow	7 (37%)	10 (32%)
Oncology attending, fellow, or NP	4 (21%)	3 (10%)

^a^ Numbers exceed 100% as multiple clinicians were often present in a discussion. Internal medicine trainees and medical students were also present in many discussions but did not contribute substantively

### Expressions of uncertainty

We identified the types of uncertainty present in the information needs that were expressed by patients and family members according to our adaptation of the uncertainty in healthcare taxonomy by Han et al. [19] (Table 2). Of the 50 discussions that contained codes for information needs, 40 had uncertainty codes (*n *= 16 for Blacks and *n = *24 for Whites). We now describe these by three main categories of uncertainty in descending order of prevalence: practical (system-centered), scientific (data-centered), and personal (patient-centered).

**Table 2:** Taxonomy of Uncertainty in Goals-of-Care Discussions (adapted from Han et al. [19])

Category	Type	Definition
Practical (system-centered)	Processes of care	Uncertainty related to practices of healthcare system and how the healthcare system operates, e.g., “should I consult with Dr. M?“
	Structures of care	Uncertainty related to healthcare system (e.g., competence of the providers)
Scientific (data-centered)	Diagnosis	Uncertainty related to understanding of diagnosis (e.g., how far the cancer has advanced)
	Causal explanations	Uncertainty that remains following causal explanations provided by the clinician regarding the person's health situation
	Treatment	Uncertainty related to treatment (e.g., how the treatment works, options that exist, whether or not they will be effective)
	Prognosis	Uncertainty related to what to expect from the course of illness
Personal (patient-centered)	Psycho-social	Effects of treatment on personal (family/friends) relationships or the welfare of one's loved ones
	Existential	Future as well as more abstract uncertainty – such as the meaning of life or concepts related to religion/faith/spirituality

#### Practical (system-centered) uncertainty

Practical (system-centered) uncertainty was the type of uncertainty most commonly expressed by all groups. Black family members frequently expressed uncertainty about processes of care, while White family members frequently expressed uncertainty about both processes and structures of care. Questions about processes of care commonly focused on logistics of care including care setting and level of support outside of the hospital, particularly in the context of pursuing a more palliative approach or enrolling in hospice:

Are we talking more hospitalizations or are you guys expecting us to pretty much deal with this at home with just pain medication? (Discussion 10, family, Black)

... would it be possible for her to stay in the hospital if she’s at a stage where she needs to stay and still have hospice here? (Discussion 27, family, Black)

Right, because what would happen is we would take her home... and then Thursday she has low blood pressure, I can’t give her blood. And we’d be running back down here again and sitting in the emergency room for four or eight hours or whatever. Just confused. (Discussion 35, family, White)

If he gets pneumonia, does he come back to the hospital or is he left with pneumonia? (Discussion 42, family, White)

Family members in particular had many additional questions specifically related to the logistics of hospice, including what level of care was provided, by whom and under whose direction, and how frequently:

But home hospice is not an option if there’s not 24-hour care, is that what you’re saying? (Discussion 9, family, Black)

Normally is that... is that the way it goes when you go to a hospice? You already have kind of like a program laid out and they’re just executing it? Or do they become responsible to develop the program? (Discussion 35, family, White)

And then how do you, would they facilitate the conversation about specifically what my Mom’s desires are? (Discussion 55, family, White)

What’s a normal amount of time the hospice nurses, when they come in, what do they usually, how long they usually stay?” (Discussion 4, family, Black)

There were also questions about cost of care whether it was expensive, and whether it was covered by insurance. There was also confusion about the definition of hospice and the common perception that hospice was for the very end of life among Black and White participants:

Hospice. I think it rings a bell, but... (Discussion 8, patient, Black)

I guess I don’t understand what hospice is. I always think it means if you are just ready to die. (Discussion 39, patient, White)

In terms of structures of care, family members, particularly White family members, expressed uncertainty about the role of palliative care as well as the distinction between palliative care and hospice:

Should palliative care be there? And if and when should hospice be involved? (Discussion 47, family, White)

So what do you do, as a palliative, what would be your role with him going home? (Discussion 13, family, Black)

Family members also expressed uncertainty as to the purpose of the discussion or the implied meaning behind what the clinician was saying, and what might have gone unsaid:

I think they’re trying to sugarcoat something they’re trying to tell us and I’m not getting it at all. I mean, I know as teachers we talk around some of the harder issues. What are you trying to say? (Discussion 20, family, White)

Because we’re having this conversation are you telling me that it’s six months or less? (Discussion 21, family, White)

When you do that six month thing, that’s a figure of speech right? (Discussion 56, patient, Black)

As can be seen, uncertainty about the purpose of the conversation seemed to reflect underlying prognostic concerns on the part of patients and family members.

#### Scientific (data-centered) uncertainty

Scientific uncertainty was the next most commonly expressed type of uncertainty and included uncertainty related to diagnosis, prognosis, causal explanations, and treatment. Few participants expressed uncertainty related to diagnosis. These statements were primarily from family members and focused on understanding the diagnosis, stage and progression of the cancer, or how the care team had arrived at their conclusions:

I didn’t know what stage the cancer was. And I have questions about the radiation... and the type of cancer... She said it’s what? (Discussion 13, family, Black)

When she went in to have the first chemo, nobody really knew what was in there so my question is how do they know? (Discussion 60, family, White)

... we have no idea how much its grown. (Discussion 14, patient, White)

Expressions of uncertainty relating to causal explanations were less frequent than other types of scientific uncertainty. Black and White patients alike encountered situations in which they did not understand treatment outcomes, particularly when outcomes did not match their expectations:

Part of what brought us in here was because she was taking pain killers that made her really groggy and we didn’t understand what the side effects would be, I think. So what we’re most afraid of is being home and having like, you know, just as simple as nausea, is that…do we take her in the hospital, don’t we? (Discussion 23, family, White)

But I just didn’t know the outcome; I didn’t know this was going to swell up. (Discussion 62, patient, Black)

Uncertainty relating to treatment was the most frequent type of scientific uncertainty expressed by both Blacks and Whites. Multiple questions focused on how treatments and medical procedures worked, treatment goals, and the availability of other treatment options:

... how do they get it in there, what, do they have an X-ray or something? (Discussion 64, family, White)

Is the medication that she on now, she won’t be taking that kind of medication that keep her knocked out all the time will she? (Discussion 28, family, Black)

So, you say a palliative care type of chemotherapy. The reason I’m doing this chemotherapy... Abraxane is really to try and improve the quality of my life? (Discussion 14, patient, White)

And so there’s a possibility that they do some lab work or whatever and then they come back and say we’re sorry you’re not a candidate for the dialysis? (Discussion 36, family, Black)

Among White but not Black patients and their family members, questions about options that had not been or were not being pursued were common:

Another question I’ve never asked the oncologist and I can’t believe I didn’t, is why was surgery never an option? Because of the metastases? (Discussion 21, family, White)

Is there any part when I even can go back on chemo again or does that...? (Discussion 60, patient, White)

At the same time, several White family members also expressed uncertainty about whether or not it was okay to forego treatment:

If, we after a week [of chemotherapy] she feels too wiped out, she doesn’t like it, can we say “No more”? (Discussion 34, family, White)

These types of uncertainty were often intermixed with concerns about future treatment and care:

What if we run into the problem that he can’t feed himself anymore? What will happen to him then? (Discussion 56, family, Black)

With regard to prognostic uncertainty, more White patients asked for quantitative prognostic information, whereas Black patients’ and family members’ expressions of uncertainty focused on how quickly disease progression might occur, what to expect from the course of illness, and how to prepare for the future:

Well, you probably can’t tell me this but how much time are we looking at? (Discussion 23, patient, White)

How fast are you expecting these things to grow? Are they going to spread to her kidney? Are they going to spread to liver? What’s going to happen? (Discussion 10, family, Black)

What’s next? How do we prepare for the worst? (Discussion 6, family, Black)

#### Personal (patient-centered) uncertainty

Expressions of personal uncertainty were less common than system and scientific-centered uncertainty, and primarily comprised existential uncertainty, the uncertainty that arises in becoming aware of one’s own mortality [35]. These statements were primarily expressed by White patients and included questions about the end of life or how they wanted it to be:

But there comes a time when I have to give up. And at that time I want to know what you people do. I don’t have experience before. I haven’t died before. (Discussion 59, patient, White)

... this is hard core, hard core. This is the reality. This is what I need to know. I want to live as vital as I can, and options that have me rolling on the floor are... not options. (Discussion 61, patient, White)

Statements of psycho-social uncertainty involved the effects of the treatment on personal (family/friends) relationships as well as on caregiver wellbeing and were uncommon: “Basically we just wanted some frame of reference of if he comes home, what it’s going to look like. What it’s going to be and what it’ll be like for him, what it’ll be like for us, and what would be the best for him.” (Discussion 49, family, White)

## Discussion

Using an adapted taxonomy of uncertainty, we found that though all uncertainty types were represented, system-centered (i.e., processes and systems of care) and data-centered (e.g., treatment, prognosis) uncertainty were most common. System-centered expressions of uncertainty focused on the definition and logistics of hospice, the role of palliative care in hospital and outpatient settings, and the distinction between palliative care and hospice. These findings are consistent with other studies reporting knowledge gaps related to palliative care and hospice [37–43].

Both Black and White family members also expressed system-centered uncertainty about how to deal with future acute medical events in the context of the patient’s progressive terminal illness if they transitioned to hospice. For many families, a shift to quality-of-life focused care, under the auspices of palliative care or hospice, introduced ambiguity about how to manage acute conditions (such as infection and bleeding) without hospital-level intervention. This focus on addressing uncertainty related to the logistics of care delivery is congruent with extant literature indicating caregivers often have unmet information needs related to nursing skills, [44] hospital processes, [45] treatment and treatment outcomes, [45] quality of care, [46] and care transitions [45, 46]. Given our findings, clinicians may want to consider explicitly exploring patients’ and families’ information needs related to the management of acute medical issues within the context of palliative care.

Certain types of uncertainty were expressed more often in the context of discussions with either Black or White patients. For example, more White patients expressed a desire for quantitative prognostic information, whereas Black family members expressed prognostic uncertainty in more qualitative terms as they sought to understand what to expect for the future. Given findings that prognostic communication occurs less frequently in palliative care consultations with Black patients compared to Whites, [28] our findings suggest there may be opportunities for clinicians to better address prognostic uncertainty for Black patients and their families particularly in the context of how to prepare for end of life.

We also found that more White patients and family members inquired about why certain treatment options were not being considered as well as whether foregoing treatment was an option. Previous research has also reported that, in outpatient settings, Black patients with cancer are less likely than White patients to actively participate in discussions with their physicians and more likely to have unmet information needs [27, 47]. Possible explanations include that White patients may have been more likely to perceive there were choices to be made and felt more comfortable asking about treatment choices. Additionally, given the racial differences in educational attainment within our sample, White patients may have felt more knowledgeable about options, and/or more empowered to raise these issues. Differences in the sense of perceived choice and control may also reflect differences in life experiences and systemic factors (e.g., structural racism).

This study has several limitations. Given the small sample size, these differences may or may not be observed in a larger sample, and we recognize the need for additional studies with larger sample sizes. Moreover, this was a qualitative study at a single academic medical center; study findings may be less generalizable to other settings. In addition, the coding occurred over multiple phases; in the second phase, we reviewed only the codes from the first phase relating to information needs. While this is congruent with our focus of identifying uncertainty underlying information needs, our results may not reflect uncertainty in goals-of-care discussions more generally.

Similarly, this study was based on actual discussions in clinical settings. The majority of the discussions in our study included a palliative care physician; thus, our findings may not reflect discussions conducted with other types of inpatient clinicians. Patients and their family may have experienced uncertainty that they did not express, and a follow-up interview study exploring these uncertainties could be an important direction for future research. Along these lines, there are times when a patient or family member might phrase something as a question, but might benefit more from an empathic, as opposed to informative, response. Future research could explore the suitability of different types of responses in context. Lastly, some time has passed since this data was collected. However, the issues that we address remain unresolved, and the findings of this study provide insight into areas for improvement in goals-of-care discussions.

## Conclusions

This study sought to characterize the types of uncertainty expressed by Black and White patients and their family members within the context of information needs during inpatient goals-of-care discussions. Using an adapted taxonomy of uncertainty in healthcare, we found that uncertainty relating to processes of care, structures of care, and treatment were most common. While racial and ethnic differences in the types of uncertainty expressed by patients and families were observed, both groups frequently expressed system-centered uncertainty, suggesting this may be an especially important target for interventions to help patients, family members, and clinicians address and manage uncertainty within the context of goals of care.

## Data Availability

De-identified transcripts are stored at the University of Washington. Data are available from the study PI, Rashmi Sharma, upon reasonable request.
